# Understanding Japan’s mortality advantage: a comparison of mortality in independent and dependent older adults in Japan and Sweden

**DOI:** 10.1186/s12916-026-04786-z

**Published:** 2026-03-23

**Authors:** Shunsuke Murata, Marcus Ebeling, Rei Ono, Megumi Maeda, Katharina Schmidt‑Mende, Haruhisa Fukuda, Karin Modig

**Affiliations:** 1 Unit of Epidemiology, Institute of Environmental Medicine, Karolinska Institutet, Box 210, Stockholm, 17177 Sweden; 2 Department of Public Health, Kobe University Graduate School of Health Sciences, Kobe, Japan; 3 Japan Society for the Promotion of Science, Tokyo, Japan; 4 Laboratory of Population Health, Max Planck Institute for Demographic Research, Rostock, Germany; 5 Max Planck – University of Helsinki Center for Social Inequalities in Population Health, Rostock, Germany and Helsinki, Finland; 6 Department of Social Science, National Center for Geriatrics and Gerontology, Aichi, Japan; 7 Center of Physical Activity Research, National Institutes of Biomedical Innovation, Health and Nutrition, Osaka, Japan; 8 Department of Health Care Administration and Management, Graduate School of Medical Sciences, Kyushu University, Fukuoka, Japan; 9 Division of Family Medicine and Primary Care, Department of Neurobiology, Care Sciences and Society, Karolinska Institutet, Huddinge, Sweden; 10 Academic Primary Health Care Centre, Stockholm Region, Stockholm, Sweden

**Keywords:** Longevity, Mortality, Long-term care, Japan, Sweden

## Abstract

**Background:**

A fundamental public health goal is that all individuals have the opportunity to reach old age with adequate care and support. Japan is the global leader in longevity, and understanding whether this advantage exists primarily in healthy older adults or those relying on long-term care (LTC) can reveal if it stems from a healthier population or more extensive, and potentially higher-quality, healthcare provision. This study examined Japan’s mortality advantage by comparing life expectancy and death rates in Japan and Sweden across different levels of LTC.

**Methods:**

We included the entire population aged 75 + in Sweden (*n* = 858,595) and nine Japanese municipalities (*n* = 334,873), categorizing individuals into three groups: no care, home care, and care home residence. We compared age-specific death rates, remaining life expectancy, and expected time spent in each LTC state. Finally, we quantified how much of the overall mortality differences could be explained by LTC state-specific mortality difference.

**Results:**

Japanese older adults had lower death rates and longer life expectancy than Swedish counterparts, with more pronounced differences among individuals utilizing LTC. At age 75, total life expectancy was 12.0 vs. 11.7 years for men and 15.5 vs. 13.7 years for women in Japan and Sweden, respectively. Expected time without LTC was 9.8 vs. 9.6 years for men and 10.4 vs. 9.9 years for women. The difference (95% CI) in total life expectancy [men, 0.3 (0.2, 0.4); women, 1.8 (1.7, 1.9)] exceeded the difference in time without LTC [men, 0.2 (0.2, 0.3); women, 0.5 (0.4, 0.5)], particularly for women. Higher mortality in home care and care home populations in Sweden substantially increased Japan’s advantage.

**Conclusions:**

Our findings show that Japan’s longevity advantage in old age is primarily driven by lower mortality in the segment of the population utilizing LTC. This indicates that the overall advantage in life expectancy may not stem solely from a healthier population, but rather from more extensive, or possibly higher-quality, care, including life-sustaining treatments. However, since we were unable to control for differences in health status in the two populations, future studies should explore if the threshold for entering LTC is different in Sweden and Japan.

**Supplementary Information:**

The online version contains supplementary material available at 10.1186/s12916-026-04786-z.

## Background

Japan stands among the global leaders in population longevity. Japanese women, in particular, have been breaking life expectancy records for several decades [1, 2]. Sweden can be considered an average European country in terms of life expectancy. Both countries have well-developed universal healthcare systems [3], but Sweden, like most European countries, lags behind Japan in terms of life expectancy, particularly for women and older adults. At age 75, a Swedish woman has a remaining life expectancy of 13.9 years, whereas a Japanese woman of the same age can expect to live 15.8 years [4], a difference of 1.9 years. Several factors have been associated with Japan’s exceptional longevity, including its healthy diet and specific cultural practices [1, 5, 6]. However, as populations around the world increasingly survive illness and reach older ages, new determinants are becoming increasingly important for life expectancy, including long-term care (LTC). The quality and extent of LTC are likely to have a substantial impact on life expectancy among older individuals.

Sweden and Japan both have well-established welfare systems that provide high-quality, low-cost health- and long-term care to their populations [3, 7]. Sweden, in particular, is internationally recognized for its comprehensive welfare support systems and its long-standing commitment to equitable LTC services [8]. Utilization of LTC, which includes instrumental, personal, and medical care at home or in institutions, may prolong survival with disease. Previous comparisons of the health status of Japanese and Swedish centenarians, conducted within the 5-COOP cohort, have revealed the proportion of frail and bedridden individuals to be higher in Japan than in Sweden [9, 10]. This invites the hypothesis that longer lifespans of Japanese individuals may be partly attributable to more extensive use of healthcare services. Supporting this interpretation, Japan has higher rates of doctor consultations and longer hospital stays compared to Sweden and many other countries [11]. By contrasting Japan with Sweden, we can gain a deeper understanding of the relationship between LTC, mortality, and longevity, which could foster our understanding of general determinants and country-specific features.

We are not aware of any population-level study that compares the proportion of individuals utilizing LTC in Japan with another European country. LTC, which is provided based on need assessment, may serve as a proxy for the degree of disability or frailty, with individuals not requiring LTC constituting a “healthy population,” individuals relying on home care constituting a “mildly or moderately disabled or frail population,” and individuals living in nursing homes constituting a “severely disabled or frail population.” Analyzing how mortality differs across age and in the healthy and disabled/unhealthy populations could provide further insights into the mechanisms behind the Japanese longevity advantage.

Therefore, using population-based longitudinal data, this study aims to better understand why Japan maintains higher life expectancy than other countries, particularly among older adults, by comparing death rates and life expectancy in Japan and Sweden across age and levels of LTC.

## Methods

### Data, study population, and measurements

The study included individuals aged 75 years and older from two population-based cohorts: the Ageing and Health Cohort (AHC) in Sweden and the LIFE cohort (The Longevity Improvement & Fair Evidence) in Japan [12]. Both cohorts draw on information from administrative health records. The AHC cohort includes the entire population in Sweden, identified through the Total Population Register. The LIFE cohort includes the entire population from 9 Japanese municipalities, including urban and rural areas [12]. One municipality is located in the Kanto area, another in the Kansai area, and seven others are located in the Kyushu area. Population sizes of the included municipalities ranged approximately from 50,000 to 1,500,000. In the Swedish data, information from several administrative registers has been linked to the individuals through the personal identification key assigned to all individuals in Sweden. Date of death, relocations, and migrations were identified using the Total Population Register in Sweden and information on LTC was extracted from the Social Service Register [13]. In the Japanese data, information from a number of data sources including date of death have been linked through Japanese Kanji name (e.g., 村田 峻輔, the first author’s Kanji name), Japanese Kana name (e.g., むらた しゅんすけ, the first author’s Kana name), sex, and birth date in each municipality. Information on date of death and relocations was obtained from residence certificate data and LTC utilization from LTC insurance. Descriptions of LTC insurance and health care insurance in both countries have been explained in more detail in previous reports [7, 14–16]. The baseline was set on April 1 st, 2017, and individuals were followed for 3 years. We also pooled the data between April 2017 and April 2020 to calculate period life expectancy.

We extracted formal LTC state and classified individuals into three groups: no care (no formal LTC), home care (home care service use), and care home (residing in a care home). Home care consists of help with daily activities and/or personal care. Detailed methods and codes to identify LTC in both countries are shown in Additional file 1: Table S1 [12].

### Statistical analysis

*In the first part of the analysis*, we used an incidence-based multistate model to estimate total and care-specific (no care, home care, and care home) life expectancy from age 75 onward [17]. The model included three transient states (no care, home care, and care home) and death as the absorbing state. Individuals could either remain in their state or transition to other care states or to death, from which no further transitions were possible. We allowed for all possible transitions among the different care states, including transitions back to states with lower care demand (see Additional file 1: Fig. S1). We calculated transition and remaining probabilities across 3-month age intervals from age 70 to 105 [18]. These narrow intervals allowed us to capture transitions occurring over short time periods, which results in more precise age-specific estimates. For example, for individuals in home care at age 80.0 years, we calculated the probability of transitioning to no care, care home, or death, as well as the probability of remaining in home care, by the time they reached age 80.25 years. We applied generalized additive models with penalized splines (P-splines) to smooth the transition probabilities [19]. Based on these probabilities across all ages and states, we derived total life expectancy and state-specific estimates. We estimated 95% confidence intervals (CI) using a percentile bootstrap method with 1000 iterations. All multistate-based calculations were performed using the DTMS package [20].

*In the second part of the analysis*, we examined how age-specific death rates varied between countries and sexes across different care levels. First, we calculated proportions of the population in each LTC state at baseline (1 month prior to the baseline month of April 2017) by dividing the number of individuals in each state by the total baseline population, separately for each country, sex, and 5-year age groups (75–79, …, 95–99, 100 + years). Age-standardized proportions and their differences between countries were also calculated. We used the Wald-based method to calculate 95% CIs for these estimates, with variance assuming Bernoulli distribution [21].

Next, we calculated age-specific death rates for 5-year age groups by dividing the number of deaths by person-years at risk over a 3-year period from baseline. We followed them for 3 years to avoid the COVID-19 pandemic, which affected Sweden and Japan differently [22, 23]. However, data from both countries are available until April 2022 and a sensitivity analysis on mortality difference was conducted by extending the follow-up period until April 2022. The death rates were calculated separately for each sex, country, and baseline LTC status. We used rate ratios and rate differences to compare death rates, which we age-standardized using the age structure of the combined cohorts from both countries, with stratification by sex and baseline LTC state. We calculated the 95% CIs for these estimates, with variance assuming Poisson distribution [21].

Finally, we investigated how differences in care-specific mortality and population composition by LTC state contributed to the overall mortality difference between the countries. To do so, we decomposed the difference in age-specific death rates of the total population into the contributions of varying care-specific mortality (no care, home care, and care home) and varying population composition in baseline LTC status. The decomposition was performed using a general demographic algorithm described in detail elsewhere [24]. Difference in the LTC distribution can be interpreted as how much age-specific proportional difference in LTC state between the countries increased total mortality difference as well as mortality difference from each LTC state can be interpreted as how much age-specific mortality difference in each LTC state increased total age-specific mortality difference. Decomposition of age-standardized mortality difference was also applied. We used the percentile bootstrap method to calculate the 95% CI.

To assess whether our data are representative, we compared the total life expectancy estimates with national total life expectancy from the Human Mortality Database [4].

All data were analyzed using R, version 4.3.1 [25], and all R codes for the analyses are available at https://github.com/shunsukemurata/jpn-swe-mortality-ltc.

## Results

Table 1 presents number, proportions, and median ages of individuals in different care states by sex and age group at baseline in Sweden and Japan, respectively. In total, the study included 858,595 individuals in Sweden and 334,873 in Japan. Both cohorts had an average follow-up time of 2.7 years with a higher share of women in Japan than in Sweden (61.4% vs. 57.5%). The total proportion receiving home care was higher in Japan than in Sweden: 10.9% vs. 10.2% for men and 18.0% vs. 16.2% for women. In contrast, a lower proportion resided in care homes in Japan compared to Sweden, 3.6% vs. 5.7% among men and 9.0% vs. 10.2% among women. Japanese men without LTC were slightly older than their Swedish counterparts, whereas for women age distributions were similar in both countries. Japanese home care users were younger than their Swedish counterparts for both sexes. The median age and age distribution were similar for care home residents in Japan and Sweden. Transition probabilities between LTC states are shown in Additional file 1: Figs. S2 and S3, providing detailed transition patterns that underly the life expectancy calculations.

**Table 1:** Number, proportions, and median ages of individuals in different care states by sex and age group at baseline in Sweden and Japan, respectively

Characteristic	No long-term care		Home care		Care home	
	Swedes	Japanese	Swedes	Japanese	Swedes	Japanese
Men						
Total, *N* (%)	306,740 (84.2%)	110,592 (85.5%)	37,044 (10.2%)	14,079 (10.9%)	20,714 (5.7%)	4607 (3.6%)
Age						
Median (Q1, Q3)	79 (77, 83)	80 (77, 83)	85 (81, 89)	84 (80, 88)	86 (81, 90)	86 (81, 90)
*N* (%)						
75–79	158,286 (51.6%)	54,658 (49.4%)	6560 (17.7%)	3240 (23.0%)	3338 (16.1%)	738 (16.0%)
80–84	90,843 (29.6%)	35,875 (32.4%)	10,152 (27.4%)	4509 (32.0%)	5315 (25.7%)	1237 (26.9%)
85–89	43,571 (14.2%)	15,307 (13.8%)	11,430 (30.9%)	4027 (28.6%)	6101 (29.5%)	1342 (29.1%)
90–94	12,368 (4.0%)	4124 (3.7%)	7013 (18.9%)	1883 (13.4%)	4376 (21.1%)	984 (21.4%)
95–99	1600 (0.5%)	577 (0.5%)	1760 (4.8%)	370 (2.6%)	1448 (7.0%)	272 (5.9%)
100 +	72 (0.02%)	51 (0.05%)	129 (0.3%)	50 (0.4%)	136 (0.7%)	34 (0.7%)
Women						
Total, *N* (%)	363,908 (73.7%)	150,026 (73.0%)	79,954 (16.2%)	37,042 (18.0%)	50,235 (10.2%)	18,527 (9.0%)
Age						
Median (Q1, Q3)	80 (77, 84)	80 (77, 84)	86 (82, 90)	85 (81, 89)	88 (84, 92)	88 (84, 92)
*N* (%)						
75–79	173,767 (47.8%)	68,692 (45.8%)	10,415 (13.0%)	5725 (15.5%)	4366 (8.7%)	1480 (8.0%)
80–84	109,139 (30.0%)	48,043 (32.0%)	19,803 (24.8%)	11,026 (29.8%)	9320 (18.6%)	3522 (19.0%)
85–89	58,655 (16.1%)	23,009 (15.3%)	26,005 (32.5%)	11,688 (31.6%)	14,922 (29.7%)	5628 (30.4%)
90–94	18,979 (5.2%)	8040 (5.4%)	18,005 (22.5%)	6456 (17.4%)	14,232 (28.3%)	5093 (27.5%)
95–99	3124 (0.9%)	1939 (1.3%)	5254 (6.6%)	1879 (5.1%)	6418 (12.8%)	2294 (12.4%)
100 +	244 (0.1%)	303 (0.2%)	472 (0.6%)	268 (0.7%)	977 (1.9%)	510 (2.8%)

*Q1*, first quartile; *Q3*, third quartile

**Fig. 1:**
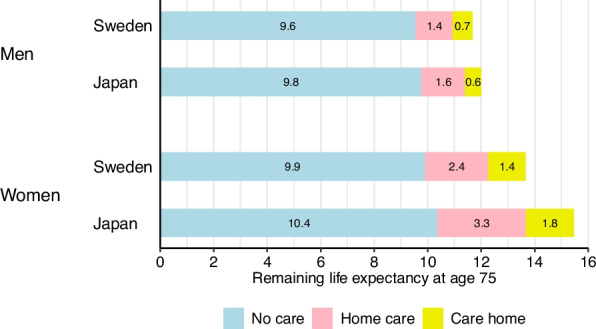
Remaining life expectancy and expected time in each care level at age 75 in Sweden and Japan, women and men, respectively

Figure 1 presents the remaining life expectancy and the average expected time spent in each LTC state at age 75 for Japan and Sweden. The lengths of the bars reflect total life expectancy, while the care-specific bars reflect the average expected time spent in each LTC state. Total life expectancy was higher in Japan than in Sweden with 12.0 years vs. 11.7 years for men and 15.5 years vs. 13.7 years for women. The difference in total life expectancy was comparable to, or slightly greater than, the difference in time spent without LTC. Japanese men spent 0.2 (95% CI, 0.2, 0.3) years longer without LTC and had 0.3 (0.2, 0.4) years longer total life expectancy than Swedish men. Among women, the gap in total life expectancy exceeded the difference in time spent without LTC. Japanese women spent 0.5 (0.4, 0.5) years longer without LTC and had 1.8 (1.7, 1.9) years longer total life expectancy than Swedish women.

**Fig. 2:**
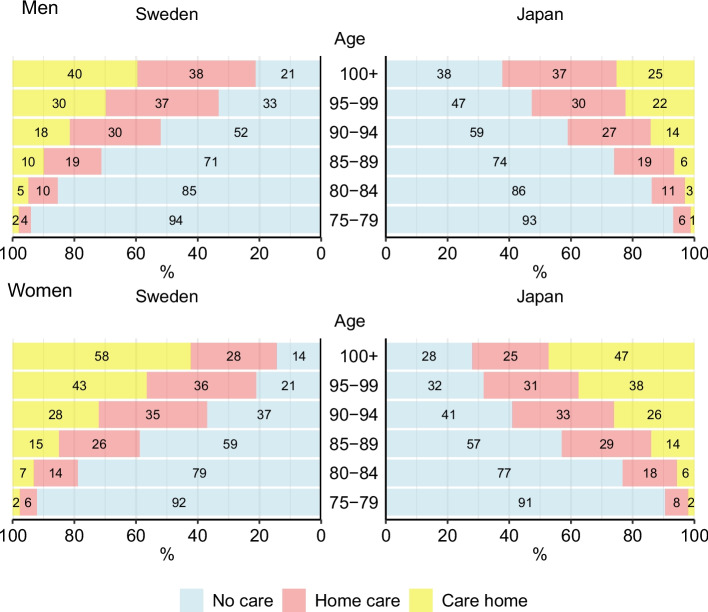
Proportion of the population in Sweden and Japan in each care level by age and sex

Figure 2 shows the age-specific proportions of the population in different LTC states by sex and country. Overall, Japanese men were less likely to use LTC than Swedish men [age-standardized proportion difference of no care (95% CI), − 1.3 (− 1.6, − 1.0)]. Among women, Swedish women had a higher proportion without LTC until age 90 and also in the total population with a difference of 1.1 percentage points (95% CI, 0.8, 1.3). The proportion of individuals receiving home care was lower in Sweden than in Japan for both sexes [men, − 0.8 (− 1.1, − 0.5); women, − 2.0 (− 2.3, − 1.8)], whereas the proportion living in care homes was higher in Sweden [men, 2.1 (1.9, 2.3); women, 1.0 (0.8, 1.2)].

**Fig. 3:**
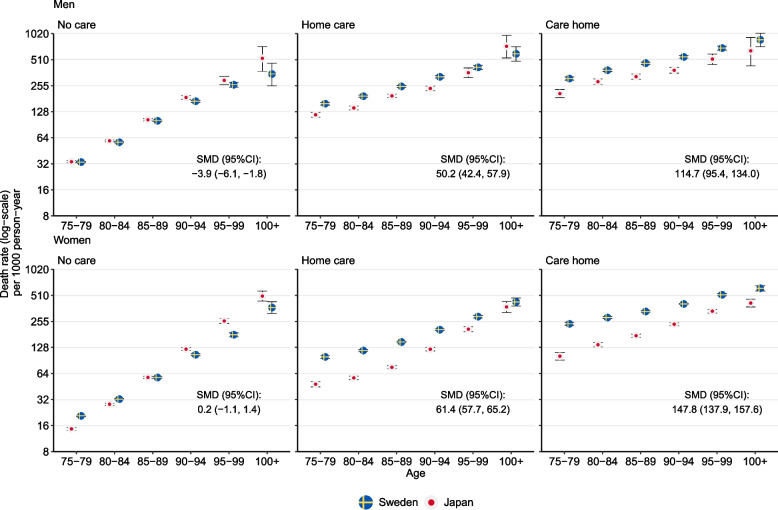
Age-specific death rates per 1000 person-years for Sweden and Japan stratified by sex and long-term care level. SMD, age-standardized mortality difference; CI, confidence interval. The error bar shows 95% CI

Figure 3 shows age-specific death rates by country, stratified by sex and care states, along with absolute differences in the age-standardized death rate combined for all ages. In both countries, death rates were substantially higher among individuals receiving LTC compared to those without LTC. Japanese older adults generally had lower death rates compared to their Swedish counterparts across nearly all subgroups, except for men without LTC. The mortality gap widened as care levels increased. In the population not receiving LTC, age-standardized death rates (95% CI) were slightly lower or similar in Sweden, with men experiencing 4 (2, 6) fewer deaths and women 0.2 (− 1, 1) more deaths per 1000 person-years. Japanese men in home care had a lower age-standardized death rate corresponding to 50 (42, 58) fewer death per 1000 person-years, and Japanese women 61 (58, 65) fewer deaths compared with their Swedish counterparts. Among care home residents, the mortality differences increased to 115 (95, 134) for men and 148 (138, 158) for women. Notably, among the very oldest individuals without care, mortality rates were higher in Japan than in Sweden. Detailed results on age-standardized mortality rate are provided in Additional file 1: Fig. S4.

**Fig. 4:**
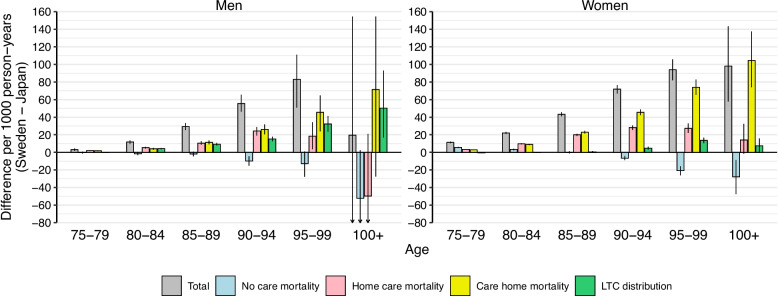
Age-specific mortality difference (deaths per 1000 person-years), and decomposition of the age-specific differences into differences in care state distribution, as well as differences in death rates within each care level. The error bar shows 95% confidence intervals

Figure 3 illustrates death rates by care states in Japan and Sweden but did not assess how care-specific mortality differences contributed to overall age-specific mortality differences. Figure 4 presents the result of decomposing the difference in total age-specific deaths rate between Japan and Sweden for men and women, respectively. Overall, death rates in the total population were lower in Japan than in Sweden across all age groups. For both men and women, Japan’s mortality advantage was driven primarily by lower mortality within home care and care home populations. This advantage was partially offset by higher mortality in Japanese without LTC, particularly at the oldest ages. For example, among women, the lower mortality in the home care population at ages 85–89 contributed to 20 fewer deaths per 1000 person-years and the corresponding number for the care home population in the same age group was 23 deaths. Overall, Japan’s lower LTC utilization also contributed to the advantage, particularly at older ages and in men. Decomposition of total (age-standardized) mortality difference is shown in Additional file 1: Fig. S5. For men, Japan’s lower mortality in home care and care home populations contributed equally to the advantage, followed by the lower LTC utilization. For women, lower mortality in care home accounted for most of the mortality difference, followed by the lower mortality in home care.

Sensitivity analyses extending follow-up through the COVID-19 pandemic period (Additional file 1: Figs. S6, S7, and S8) yielded consistent results, with care-specific mortality differences remaining the primary driver of overall mortality differences between countries.

Our estimates were similar to national estimates though Japanese data underestimated them slightly (Additional file 1: Fig. S9, maximum difference was 0.4 years).

## Discussion

This study aimed to better understand why Japan maintains higher life expectancy than other countries, particularly among older adults, by comparing death rates and life expectancy in Japan and Sweden across age and levels of LTC. The findings showed that a larger proportion of Swedish men utilized LTC compared with men in Japan. Among women, this difference became evident from around age 90. Furthermore, older adults receiving LTC in Japan experienced lower mortality at almost all ages compared with their Swedish counterparts. These mortality differences, and the associated differences in expected time spent in each LTC state, became more pronounced with advancing age and increasing care needs. In contrast, mortality did not differ substantially between the two countries among individuals not receiving LTC.

Several factors may explain these findings, and we discuss some of them below. If we assume that the need-assessment processes for LTC are broadly comparable in Japan and Sweden, the results suggest that Japan’s mortality advantage does not stem from a generally healthier older population, but rather from lower mortality among those receiving LTC. This could reflect more intensive, or possibly higher-quality, care.

However, it is also possible that Japan’s threshold for entry into formal LTC is lower compared to Sweden. In that case, Japanese LTC recipients would represent a comparatively healthier group, partly explaining their lower mortality. This interpretation is partly supported by the slightly younger age of Japanese home care recipients in our data and also in previous comparative research [26]. However, the earlier study did not find major differences in overall frailty between the two LTC populations, although some variation in ADL (activities of daily living) difficulties was observed: Swedish individuals had more difficulties with bathing and dressing, whereas Japanese individuals reported more difficulties with toileting, mobility, and transfers [26, 27]. Notably, among those with severe care needs, who require formal LTC service for approximately 12 h per week or more, Japanese individuals actually demonstrated greater ADL and mobility limitations. This argues against a systematically lower threshold for LTC entry in Japan. Nevertheless, that earlier research was based on small samples and data collected nearly 15 years before our study period, which limits direct comparability.

The threshold for entry into LTC is shaped by a range of institutional, cultural, and financial factors in both countries. Conversely, some determinants could contribute to a higher threshold of LTC entry in Japan. Japan employs a standardized national assessment system that allocates individuals to one of seven care-need levels based largely on functional disabilities. In contrast, Sweden uses a decentralized system at the municipality level and guided by the Social Services Act, in which need assessments are centered on an individual’s inability to manage independently. Both countries provide substantial financial support for LTC but the proportion of expenses paid out-of-pocket is higher in Japan than in Sweden. This could increase the incentives for informal care provision, which is more common in Japan than in Sweden [28]. If informal care delays entry into formal LTC, the “no care” group in Japan may thus contain a larger share of individuals with care needs. This might also explain the partially higher death rates in the Japanese no care group. Moreover, living alone is more common in Sweden than in Japan [29], and previous research in Sweden shows that living alone is one of the strongest predictors for receiving formal LTC after age and health status [30]. This likely contributes to both a higher share receiving home care and earlier entry, supporting the hypothesis that informal care plays a role for the difference in the entry to formal LTC in both countries. Taken together, these factors suggest that in Sweden, individuals in each of the three LTC states may be more heterogeneous in terms of health status and functional limitations, whereas in Japan, the boundaries between LTC states may be more blurred due to the greater role of informal care prior to formal LTC entry.

Beyond factors affecting LTC entry, differences in healthcare provision may also contribute to Japan’s mortality advantage among LTC recipients. In Japan, cultural values have traditionally emphasized family-oriented end-of-life decision-making [31], although the situation may be changing. Therefore, Japan may provide more active and extensive end-of-life care than other nations. International comparisons show that Japan provides some of the most extensive medical care globally, while Sweden ranks lower on indicators such as hospital length of stay and medical doctor consultations [11]. Japan also has one of the highest proportions of in-hospital deaths globally [11]. While these indicators may reflect a range of systemic and cultural factors, they may also suggest a stronger emphasis on extensive healthcare for older adults in Japan, including active end-of-life care, which contributes to longer survival among individuals receiving LTC. However, whether this life extension is accompanied by maintained quality of life and aligns with patient preferences remains an important question that our data cannot address.

A key objective of public health is to ensure that all individuals have the opportunity to reach old age, rather than enabling a selected few to achieve exceptional longevity. Both Sweden and Japan have largely achieved this, yet a substantial difference in remaining life expectancy at age 75 remains. Our findings suggest that if Sweden seeks to further increase the remaining life expectancy at older ages, attention may need to shift toward the LTC population. Although Swedish healthcare performs well internationally, especially in cancer and cardiovascular care [3], Sweden does not rank among the top countries in old-age mortality [32]. Japan, by contrast, consistently ranks at the top, especially for women. Japan may provide more life-sustaining treatments at advanced ages. However, such practices raise ethical concerns about prolonged medical intervention versus quality of life at the end of life. Care for older individuals should not focus solely on extensive and life-saving treatment but also prioritize the promotion of well-being and dignity in later life. A balanced approach should thus consider both life extension and the promotion of comfort, dignity, and autonomy in later life.

Several limitations should be considered when interpreting our findings. First, the Japanese data did not include the entire national population but selected municipalities. However, these municipalities included all residents, both institutionalized and community-dwelling individuals and covered both urban and rural areas. Their representativeness is supported by close alignment with national life expectancy data from the Human Mortality Database [4]. Second, we lacked data on informal care. Informal caregiving is important in both countries and may be particularly influential in Japan [28, 29]. Unfortunately, reliable data on informal care is inherently difficult to obtain. While our restriction to formal LTC ensures high specificity in identifying individuals with documented needs, some individuals classified as not receiving formal care, especially in Japan, may have substantial needs met informally. We addressed this possibility in our interpretation.

## Conclusions

A 75-year-old Japanese woman can expect 10.4 years without formal LTC and 5.1 years with LTC, compared with 9.9 and 3.8 years for a Swedish woman. These differences are primarily driven by lower mortality among Japanese LTC recipients. Differences in LTC utilization patterns also contributed to widening the gap, especially among men. Japan’s longevity advantage may thus not stem solely from a healthier population, but also from more extensive, or perhaps higher-quality, healthcare and LTC provision, including life-sustaining treatments. Variation in the threshold for LTC entry and differences in informal care provision may also explain some of the country differences. From a policy perspective, strategies to extend life expectancy at older ages must focus not only on prevention and healthy aging but also on ensuring high-quality care for frail older adults. Importantly, policies must balance life extension with quality of life, ensuring that care aligns with older adults’ values and preferences.

## Supplementary Information

Additional file 1. Table S1 Variable definitions. Figure S1 Possible transition of long-term care states and death in the multistate model. Figure S2 Smoothed probability of each transition or remaining state. Figure S3 Observed probability of each transition or remaining state. Figure S4 Age-standardized death rates and their ratios and differences between the countries. Figure S5 Age-standardized mortality difference and its decomposition. Figure S6 Age-specific death rates with 5-year follow-up. Figure S7 Age-specific mortality difference and its decomposition with 5-year follow-up. Figure S8 Age-standardized mortality difference and its decomposition with 5-year follow-up. Figure S9 Life expectancy in our study and the national data.

## Data Availability

Individual-level data from this study cannot be made publicly available due to privacy regulations and contractual restrictions. Specifically, the data are subject to the General Data Protection Regulation (GDPR) as implemented in Sweden, and our data use agreements with Japanese municipalities.

## Abbreviations

LTC: Long-term care
ADL: Activities of daily living
