# Supplementary material for: Understanding Japan’s mortality advantage: a comparison of mortality in independent and dependent older adults in Japan and Sweden
Source: BMC Med. 2026 Mar 23;24:160. doi: 10.1186/s12916-026-04786-z (PMC13007366; doi:10.1186/s12916-026-04786-z)
Supplement: Supplementary file 1 — Additional file 1. Table S1 Variable definitions. Figure S1 Possible transition of long-term care states and death in the multistate model. Figure S2 Smoothed probability of each transition or remaining state. Figure S3 Observed probability of each transition or remaining state. Figure S4 Age-standardized death rates and their ratios and differences between the countries. Figure S5 Age-standardized mortality difference and its decomposition. Figure S6 Age-specific death rates with 5-year follow-up. Figure S7 Age-specific mortality difference and its decomposition with 5-year follow-up. Figure S8 Age-standardized mortality difference and its decomposition with 5-year follow-up. Figure S9 Life expectancy in our study and the national data. [file 12916_2026_4786_MOESM1_ESM.docx]

Table S1. Variable definitions in Sweden and Japan

| Variable | Source | Procedure | |
| --- | --- | --- | --- |
| Long-term care state | Sweden, Social Service Register | Care home | BOFORM equals to 2 |
|  |  | Home care | HTJ equals to 1 and HTJTIM is more than 0 and less than 777 |
|  | Japan, Long-term care insurance D1 table (what service were used) | Care home | d1_09 equals to 32, 33, 35, 36, 37, 51, 52, 53, 54, 55, or 59 |
|  |  | Home care | d1_09 equals to 11, 12, 15, 61, 62, 65, 71-78, A1-9, or AA |


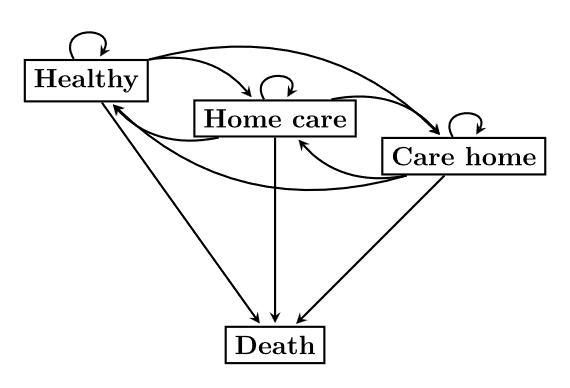
Figure S1. Possible transition of long-term care states and death in the multistate model.


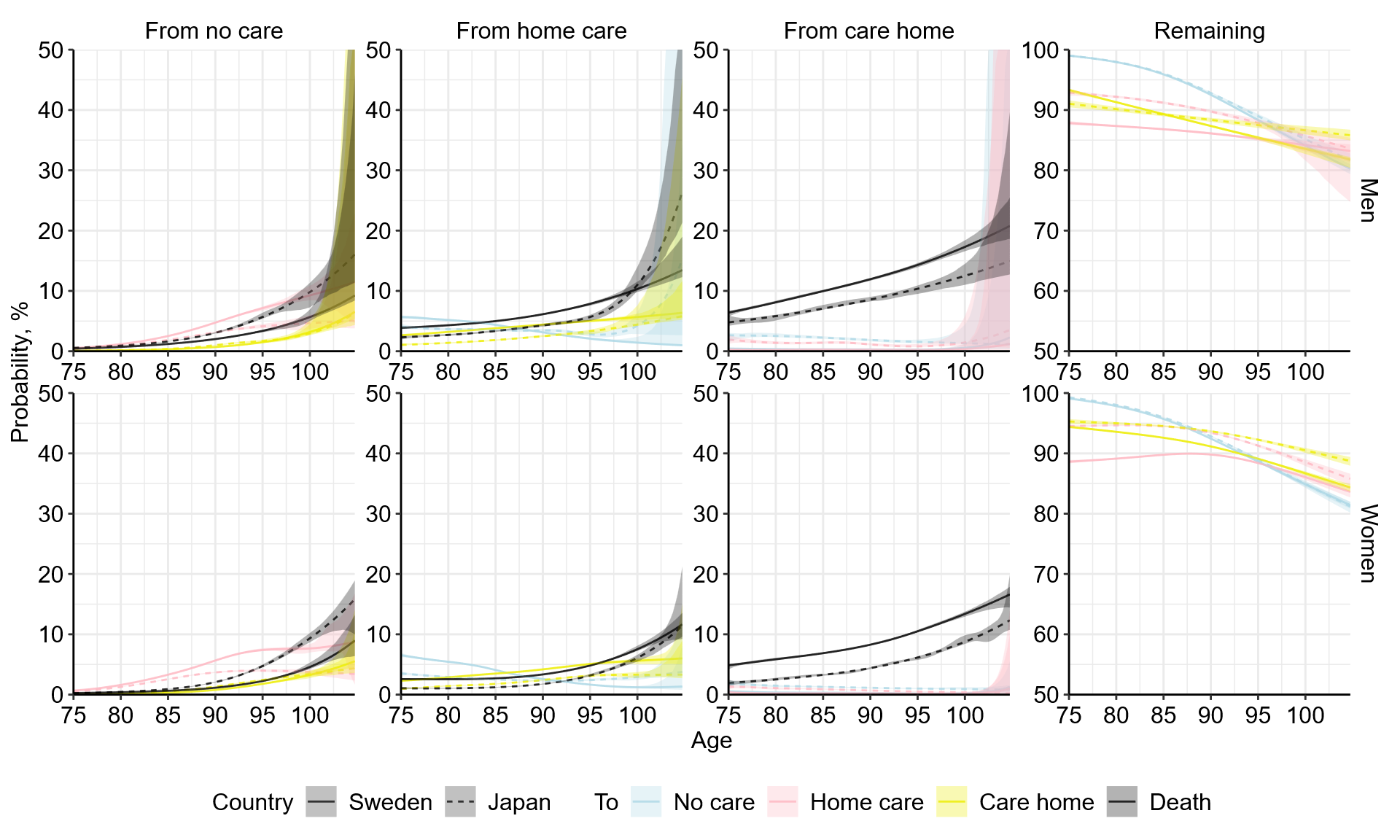
Figure S2. Smoothed probability of each transition or remaining state by age.


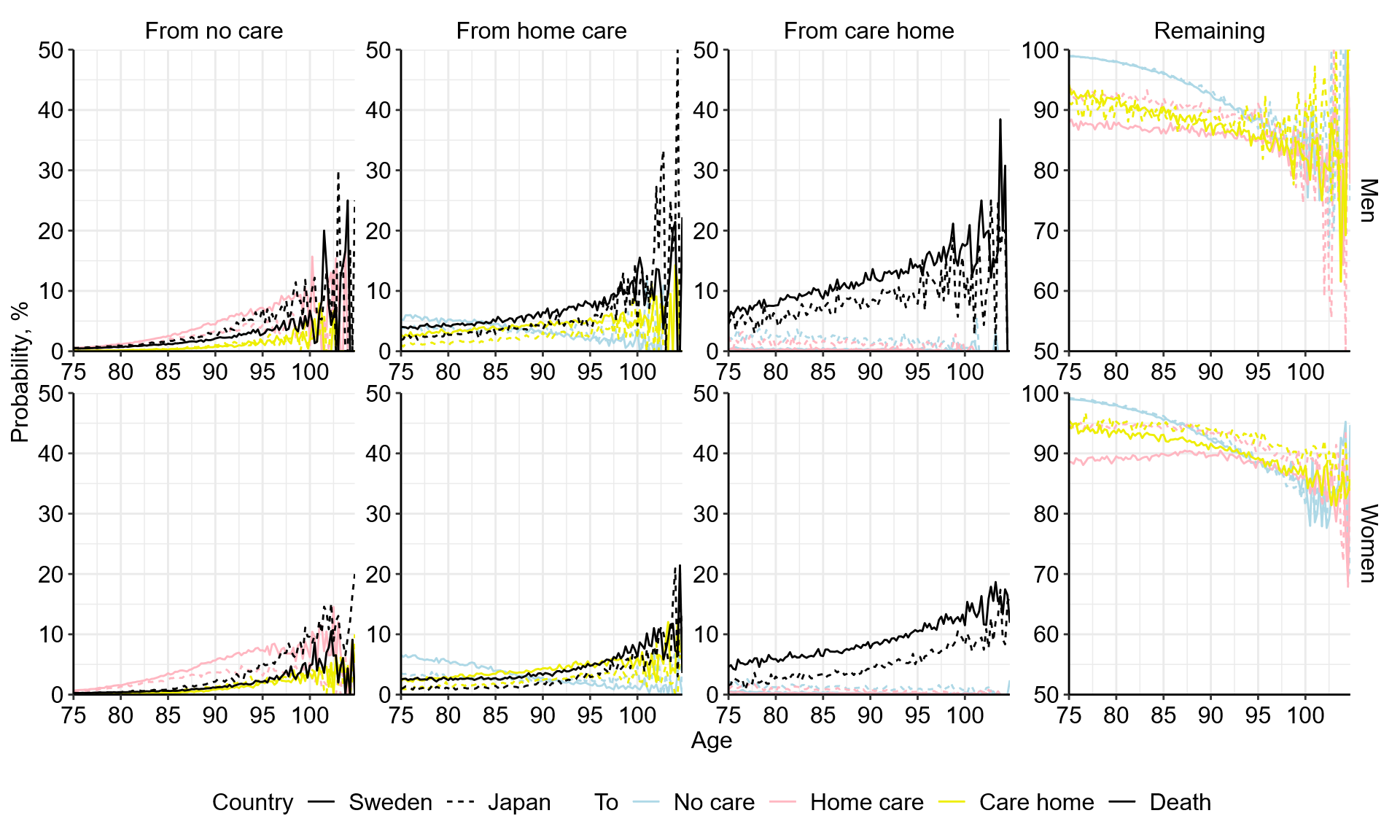
Figure S3. Observed probability of each transition or remaining state by age.


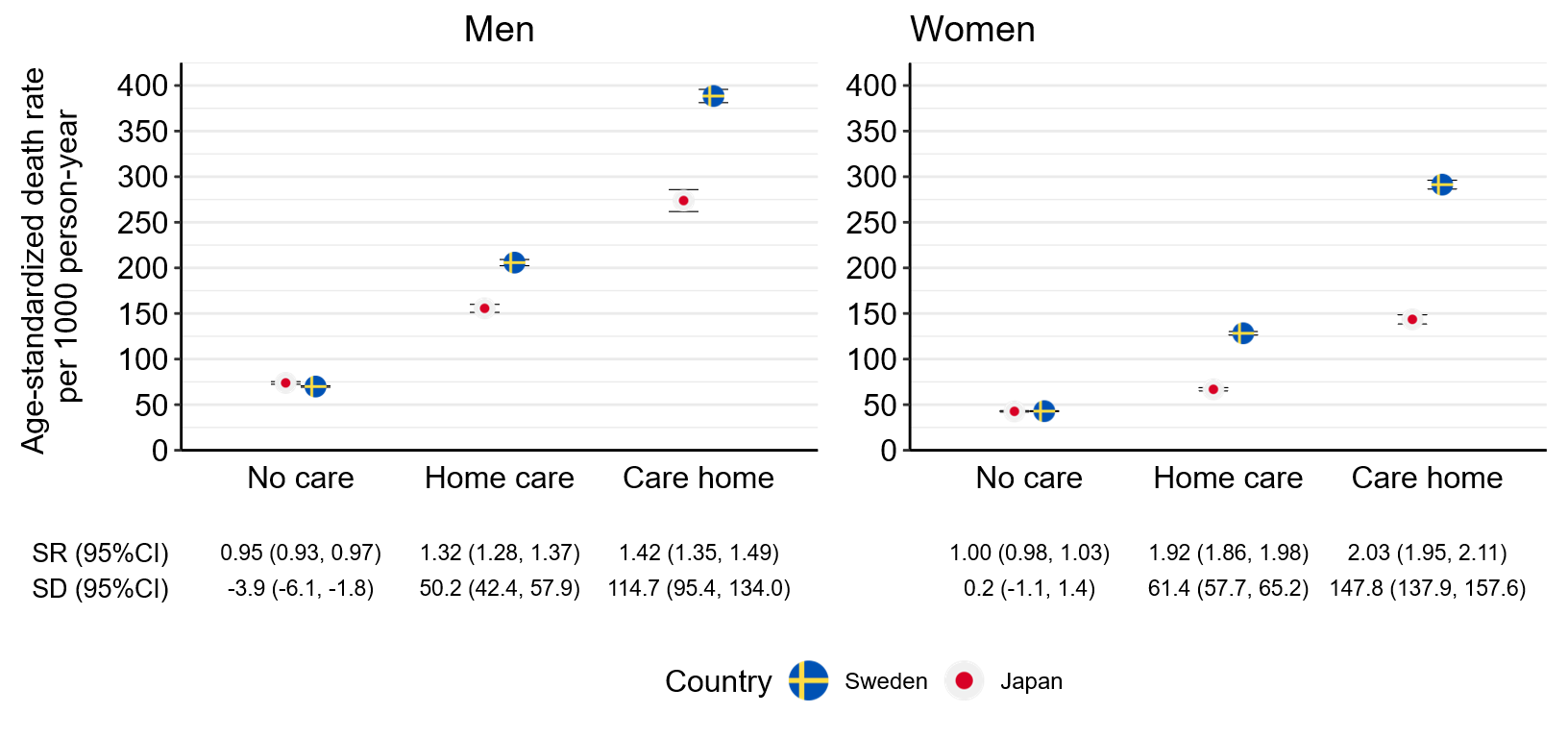
Figure S4. Age-standardized death rates per 1000 person-years for the two countries stratified by sex and long-term care states and their ratios and differences between the countries within each subgroup.

SR, Standardized ratio; SD, Standardized difference. The error bar shows 95% confidence intervals (CI).


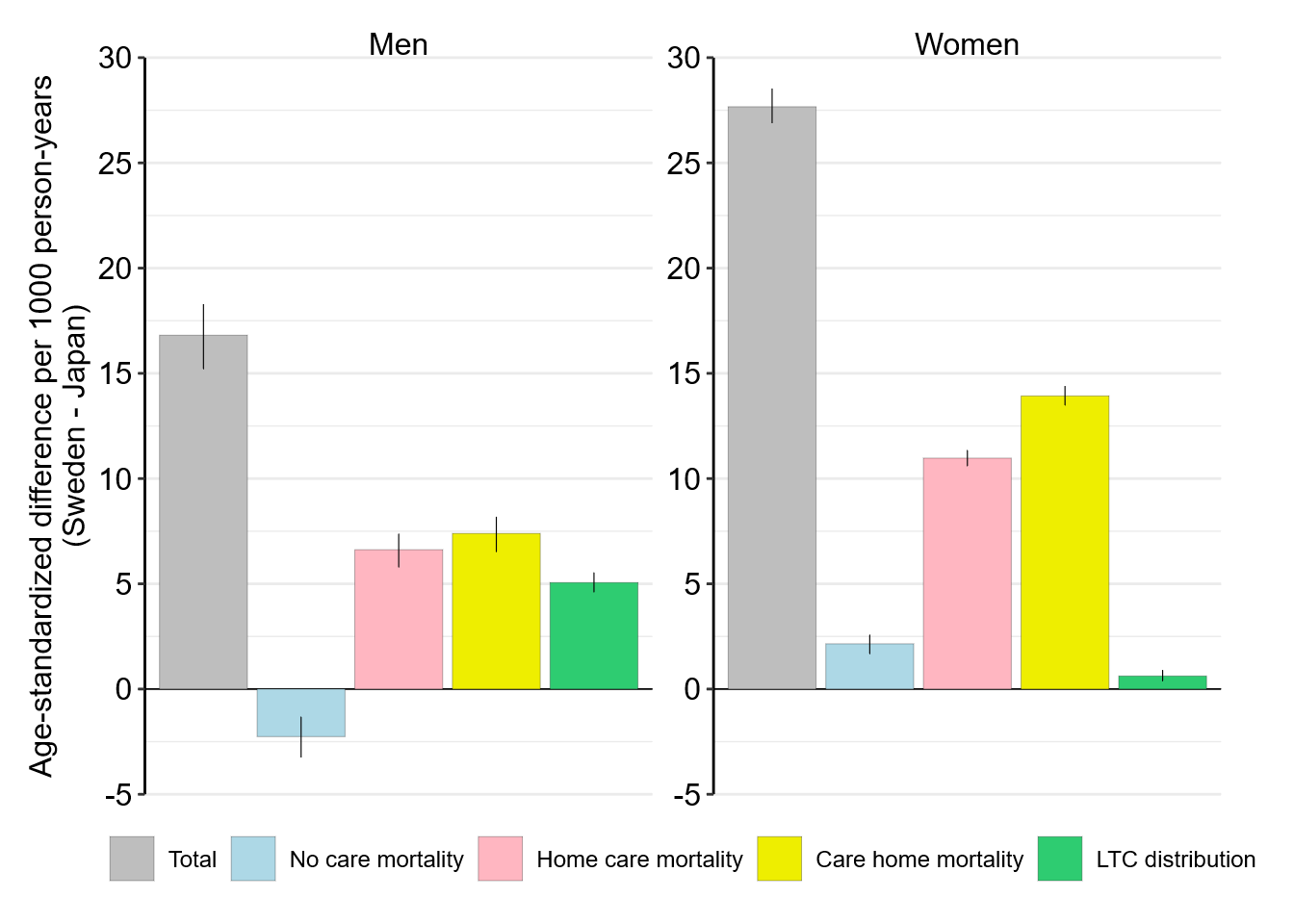


Figure S5. Age-standardized mortality difference, and decomposition of the age-standardized difference into different distribution of care states, as well as differences in death rates within each care state. The error bar shows 95% confidence intervals.


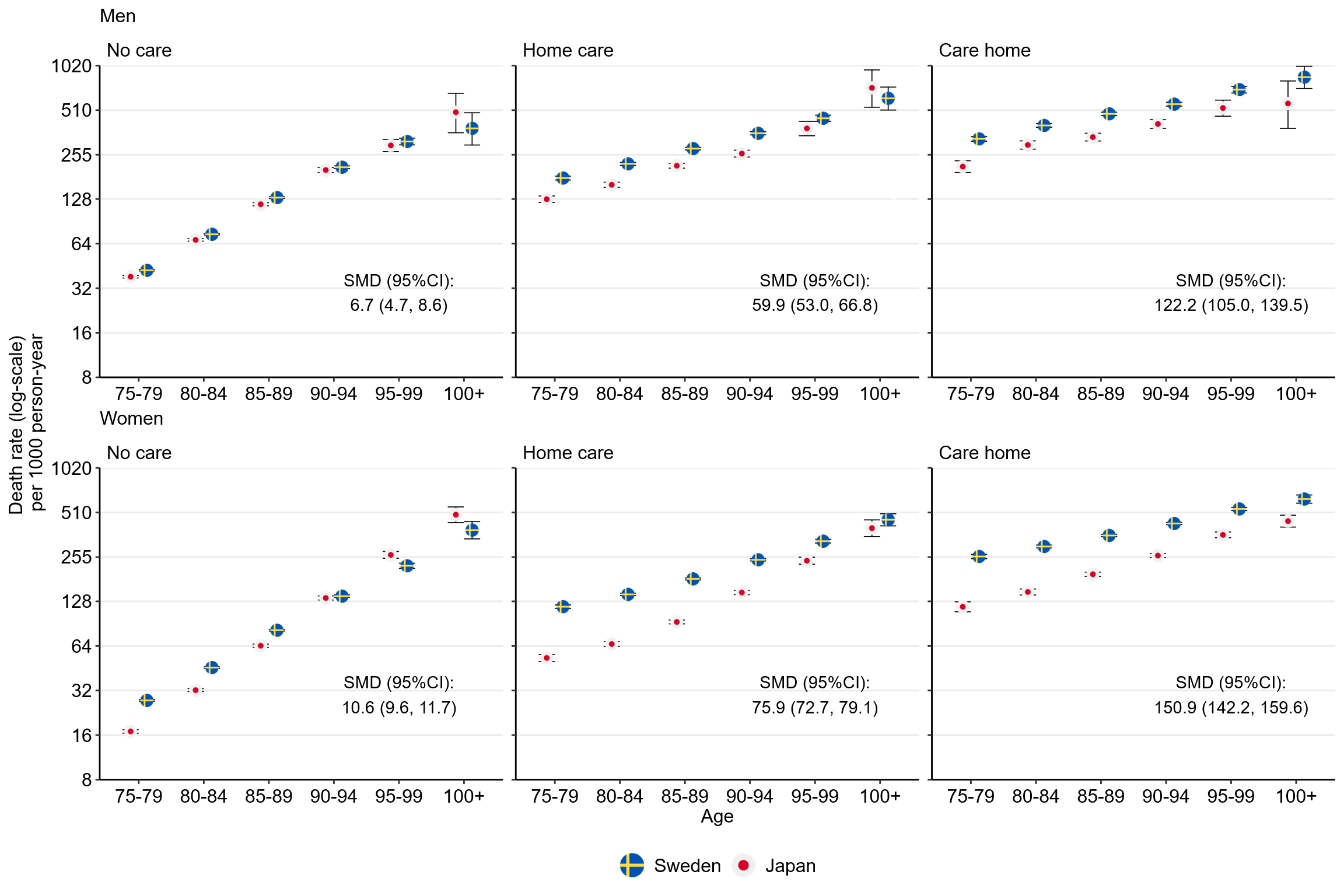
Figure S6. Age-specific death rates per 1000 person-years for the two countries stratified by sex and long-term care states with 5 years follow up until 2022, i.e., including COVID-19 pandemic.

SMD, Age-standardized mortality difference; CI, confidence interval. The error bar shows 95%CI.
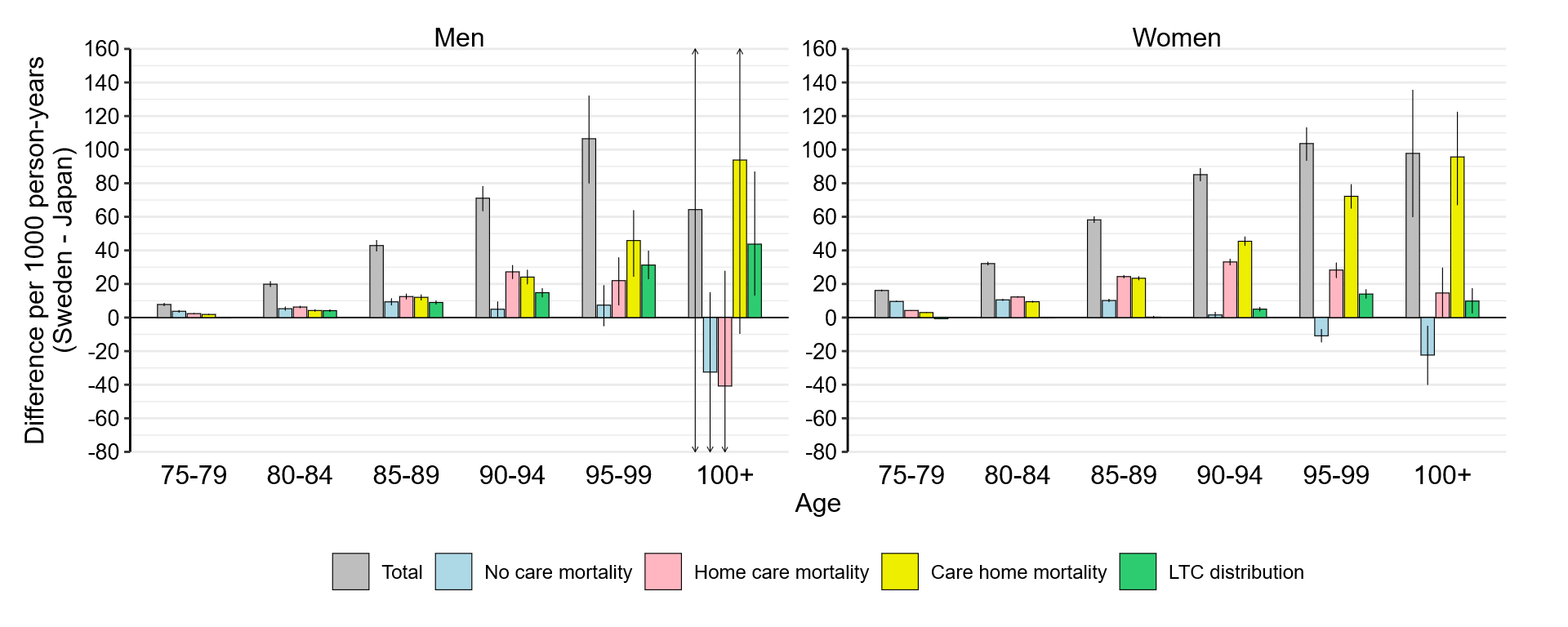
Figure S7. Age-specific mortality difference, and decomposition of the age-specific difference into different distribution of care states, as well as differences in death rates within each care state with 5 years follow up. The error bar shows 95% confidence intervals.


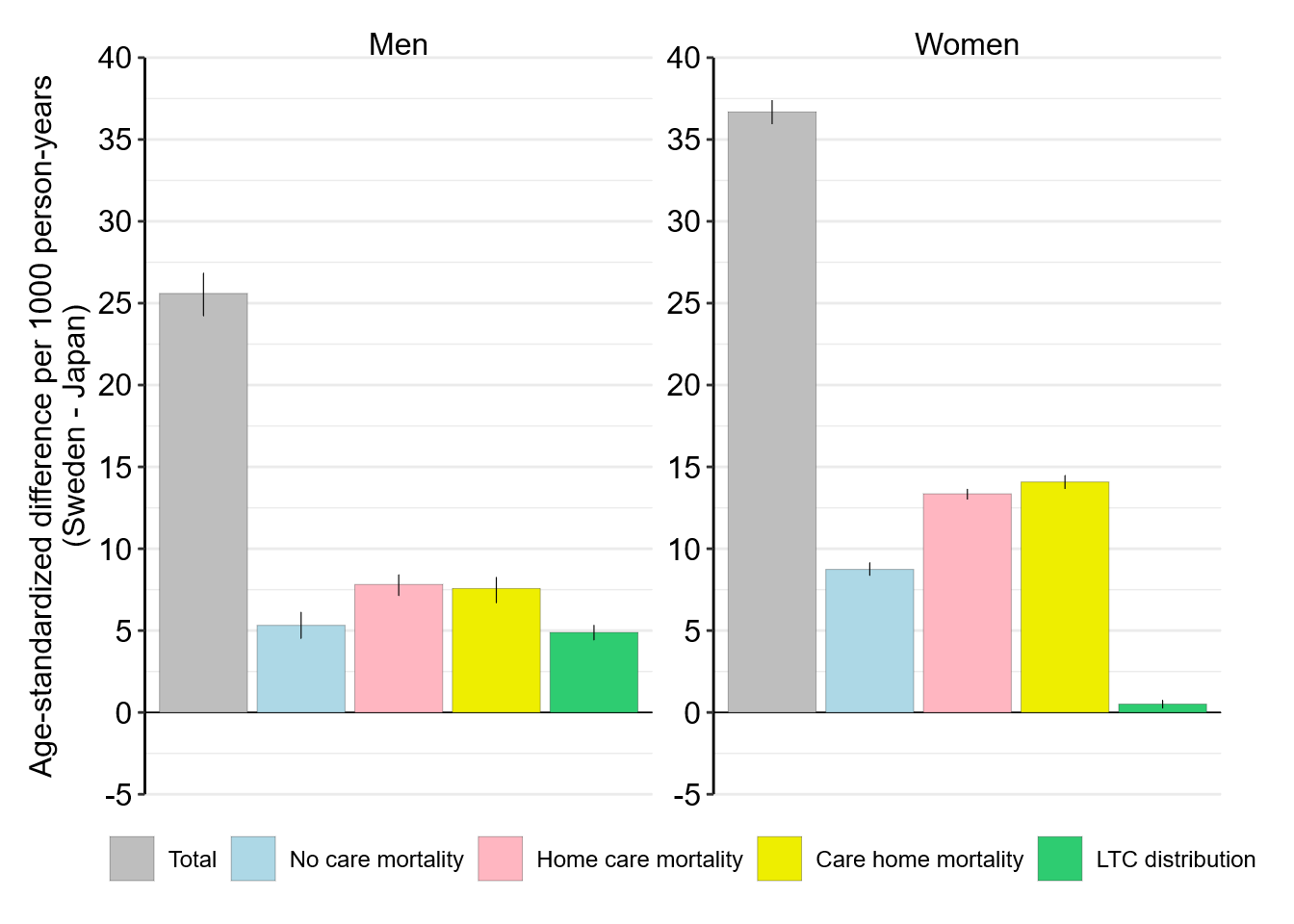


Figure S8. Age-standardized mortality difference, and decomposition of the age-standardized difference into different distribution of care states, as well as differences in death rates within each care state with 5 years follow up. The error bar shows 95% confidence intervals.


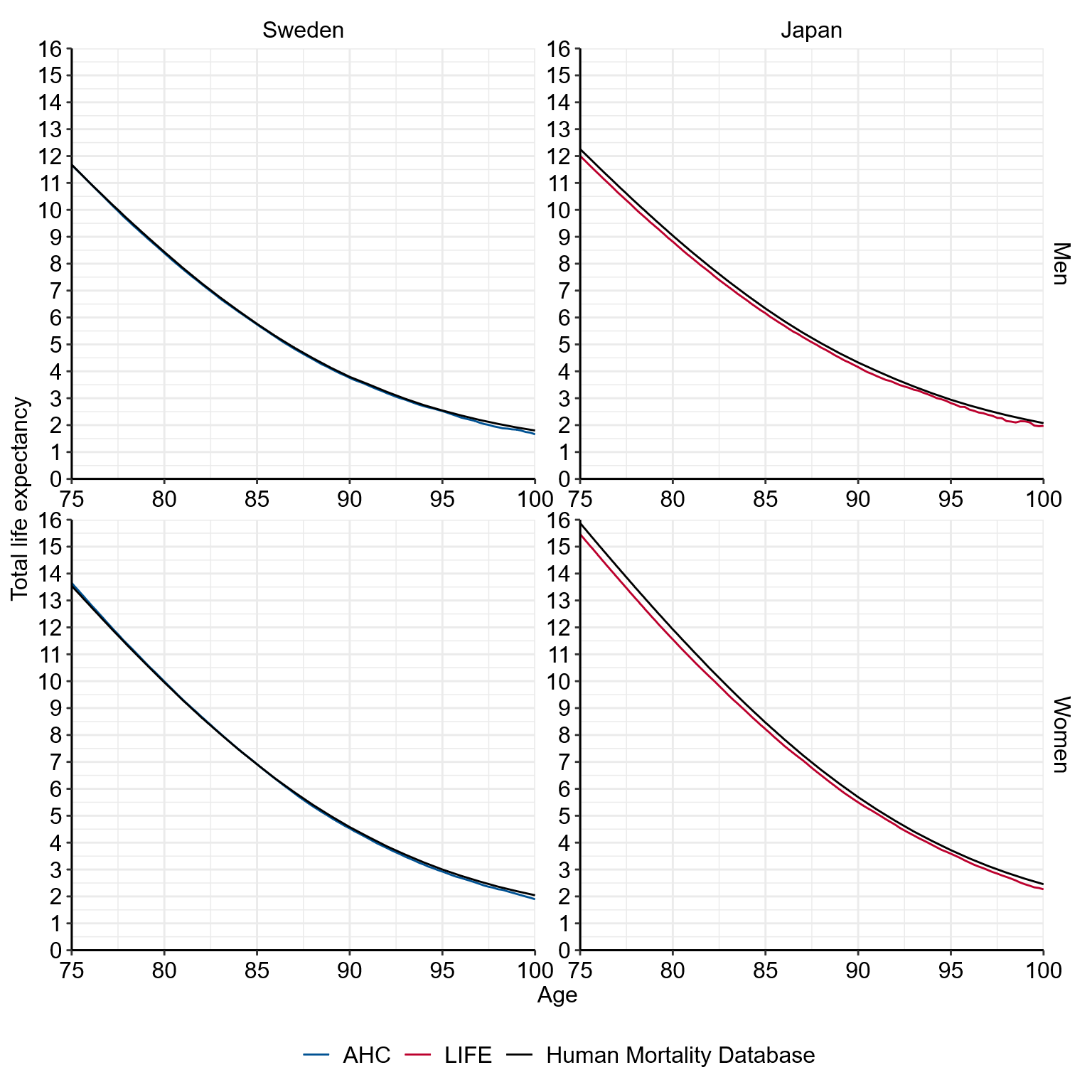


Figure S9. Life expectancy at each age in our study and the national data.

AHC, Ageing and Health cohort; LIFE, The Longevity Improvement & Fair Evidence.

The Human Mortality Database provides life expectancy data at the national level.
